# Real-world, anti-tobacco environmental impact upon price-induced smoking reduction among urban Chinese men: Evidence from China’s 2015 cigarette tax increase

**DOI:** 10.18332/tid/170596

**Published:** 2023-10-06

**Authors:** Tong Pei, Pauline Barnett, Tingzhong Yang, Ian R. H. Rockett, Weifang Zhang

**Affiliations:** 1 School of Humanities and Management, Zhejiang Chinese Medical University, Hangzhou, China; 2 School of Health Sciences, University of Canterbury, Christchurch, New Zealand; 3 Women’s Hospital, Center for Tobacco Control Research, School of Medicine, Zhejiang University, Hangzhou, China; 4 Injury Control Research Center, West Virginia University, Morgantown, United States; 5 Department of Epidemiology and Biostatistics, School of Public Health, West Virginia University, Morgantown, United States; 6 Department of Psychiatry, University of Rochester Medical Center, Rochester, United States; 7 Key Laboratory of Oral Biomedical Research, Hospital of Stomatology, School of Medicine, Zhejiang University, Hangzhou, China

**Keywords:** cigarette tax, smoking reduction, anti-tobacco environmental impact, China

## Abstract

**INTRODUCTION:**

Raising the price of cigarettes via taxation has been promoted by the World Health Organization as an important tobacco control strategy. Price elasticity of cigarettes is not uniform and is dependent upon individual and environmental determinants. Many studies have examined the determinants of price-induced smoking, taking into account sociodemographic characteristics and consumption patterns. Little research has been conducted on the association between anti-smoking environments and price-induced smoking behavior. This study addresses the deficit within the Chinese context.

**METHODS:**

Participants were 2852 male smokers identified through a multi-stage survey sampling process encompassing 6 cities in China between July and December 2016. A standardized questionnaire tapped price-induced smoking reduction and related information. Both unadjusted and adjusted logistic regression methods were applied in the analyses.

**RESULTS:**

In all, 25.5% (95% CI: 22.5–27.9) of smokers in this study decreased their smoking expenditures following the 2015 excise tax increase. The adjusted logistic regression analysis showed that increased exposures to an anti-smoking information environment (AOR=1.39; 95% CI: 1.10–1.79), restricted smoking in their home (AOR=1.67; 95% CI: 1.32–2.08) and workplace (AOR=1.43; 95% CI: 1.09–1.85) were more likely to report diminished cigarette smoking following the tax increases.

**CONCLUSIONS:**

This study adds to understanding price-induced smoking behavior among urban male Chinese smokers. Strengthening of excise tax policies needs to intensify environmental smoking restrictions and public education campaigns to increase the sensitivity of cigarette price changes among smokers.

## INTRODUCTION

Raising excise taxes on tobacco is a one of the most effective tobacco control strategies proposed by the World Health Organization^1^. There is an enormous literature that has been devoted to studying the price elasticity of demand for cigarettes^2-6^. This literature has taken advantage of the fact that smokers may modify their tobacco consumption in response to price^7,8^. The price elasticity of cigarette consumption in lower income countries is greater than in higher income countries^6,9^. Several factors appear to influence price-induced smoking reduction (PSR). For instance, low initial smoking and income levels, addiction status and a low education level might heighten price sensitivity^10-14^. Although different Asian countries vary in their political and social contexts and tax revenue policies, they share some commonalities. One study analyzed price differentials of tobacco products in 79 countries^15^. The researchers found that although some WHO South-East Asia Region (SEAR) countries have sought to raise the price of tobacco products, their tobacco control policies have not kept pace with rapid economic growth. Consequently, to reduce the affordability and consumption of tobacco products, it is important to increase taxes on tobacco products and raise their prices sufficiently to outweigh the effects of income growth^16^. Only when recognizing the high economic burden incurred by smoking do smokers quit in order to enjoy better health and more disposable income^17^.

However, sociodemographic characteristics or patterns of consumption have often been ignored^10,12-14^. Moreover, few studies examined the outcomes of anti-smoking environmental influence upon PSR. Exploring the interaction between anti-tobacco environments and PSR may reveal how PSR varies under different tobacco control situations. Hence, it is imperative to understand how an anti-tobacco environment heightens smoker sensitivity to an increase in excise taxes, and in so doing helps policymakers decide what tax rate is needed to meet smoking prevalence targets.

There is abundant evidence on the effectiveness of an anti-tobacco environment in reducing cigarette consumption^18^. An important strategy promoted by the World Health Organization has been price increases effected through taxation, with the most recent overview of systematic reviews of the effectiveness of tobacco control policies recommending prioritization of smoking bans as well as price increases^18^. The effectiveness of smoking bans in moving perceptions of smoking from ‘normal’ to ‘non-normal’ is apparent from migrant studies^19^. Bans create a supportive environment for quitting^20^. We hypothesize that smoking bans in the home and the workplace are associated with PSR.

Information–motivation–behavioral skills theory argues that anti-tobacco information depresses the desire to smoke^21^. Risk health awareness of smokers from exposure to anti-tobacco information environments may increase the sensitivity of cigarette pricing. Together with other measures, such as environmental anti-smoking information, smoking bans reduce cigarette consumption at particular sites, and promote a pervasive community view that smoking is both undesirable and unacceptable^21^. One study indicates that an anti-tobacco information environment, in concert with smoking restrictions, is associated with both attempts to quit and successful quitting among adult males^22^. A second hypothesis in our study is that environmental anti-tobacco information from public health sources is associated with PSR.

The Chinese Ministry of Finance (MOF) and the State Tobacco Monopoly Administration (STMA) raised the tax on cigarettes in May 2015. The average retail price of a pack of cigarettes in China increased about 1 Chinese Renminbi (about US$0.15) per pack, from 11.74 to 12.82 RMB, following this increase. After the tax increase, the excise tax accounted for 36.3% of the total Chinese cigarette price^14^. The main aim of this study was to examine the impact of anti-smoking information and smoking bans in the home and workplace on PSR among urban male smokers in China following the 2015 tax increase.

## METHODS

### Study area and participants

This was an observational cross-sectional, multilevel study with a multi-staged cluster sampling design. Six cities were selected from across China, and they were differentiated by region: Northeast (Jilin), North Central (Taiyuan), Northwest (Xianyang), Southwest (Chongqing), Southeast (Hangzhou), and South Central (Guangzhou). There are also differences in the regional cultural and economic developmental levels of these cities. Jilin has a population of 4.1 million and a per capita Gross Domestic Product (GDP) of 34355 RMB. Taiyuan is the capital of Shanxi province and has a population of 3.8 million and a per capita GDP of 90698 RMB. Both Jilin and Taiyuan are manufacturing cities. Xianyang (4.6 million populations and a per capita GDP of 50338 RMB) is characterized by agriculture and light industry. Chongqing is a municipality directly under the Central Government, with a population of 34.1 million and a GDP of 5828 RMB. It is dominated by agriculture and light industry. Hangzhou is the capital of Zhejiang province, and has a population of 6.9 million and a GDP of 152465 RMB. It features light industry and tourism. Guangzhou is the capital of Guangdong province and has a population of 9.4 million and a GDP of 156427 RMB. It is characterized by light industry and commercial development^23^. Within each of the six cities in this study, two residential districts were randomly selected from the main urban zones, and four communities within each district. The family household registration (‘hukou’) list was used to randomly sample households within each community.

Our sample was limited to males aged ≥15 years, who had resided in the selected cities for at least one year^22^. If there were two or more male residents in a household, the one whose birth date fell closest to the date of contact was selected to be surveyed.

### Data collection

The study was conducted during the period July to December 2016. Once an individual was identified and agreed to participate in the survey, a self-administered questionnaire was distributed to him/her. All the researchers were fourth-year graduate students in medicine and received a one-day training on the study protocol and interviewing procedures^22^. Verbal consent was obtained from all respondents, following an introduction of the questionnaire’s aims from a researcher. As appropriate, a token of appreciation (such as soap and toothbrush, valued at approximately 10 RMB) was given to participants following survey completion. The same survey protocol was used across the six cities to ensure consistency between interviews and data collection. The study was approved by the Ethics Committee at the Zhejiang University Medical College.

### Measures

*Dependent variable*

The dependent variable was smoking reduction in response to the 2015 cigarette price increases (i.e. Price-induced Smoking Reduction: PSR). Current smokers were asked: ‘Did you reduce your cigarette consumption due to the cigarette price increase in 2015?’. The response options were: ‘no reduction; a small reduction; moderate reduction; large reduction; and quit smoking’^24^. For analysis, responses were summarized under two categories: No reduction and reduction (combines all ‘reduction’ response options).

*Independent variables*

The presence of an anti-smoking information environment was measured by the question that asked respondents whether they had seen any anti-tobacco advertisements or related information in their city during their work, leisure, and social activities in the previous 6 months. Response categories were: ‘never’, ‘once’, ‘twice’, ‘three times’, or ‘four or more times’. Environmental anti-smoking information refers to the environmental atmosphere formed by various public education initiatives on tobacco control involving diverse media and formats^25,26^.

The two other independent variables pertaining to the anti-smoking environment addressed smoking restrictions in both the household and workplace. Respective responses were differentiated as: ‘none’, ‘partial’, or ‘complete’. For retired or unemployed respondents, ‘workplace’ referenced the location where they went for temporary work, leisure, or community activities. For students, ‘workplace’ covered classrooms and libraries, and the ‘household’ environment referred to dormitories^22^.

*Control variables*

Personal control variables hypothesized as influencing the purchasing response to an increase in the price of cigarettes were differentiated as: demographic/socioeconomic, smoking status and practice, and health status.

Demographic/socioeconomic characteristics tapped in the survey were age, ethnicity, education level, occupation, marital status, and household income. As previously noted, these characteristics have been shown to vary with purchasing decisions relative to cigarette price^27,28^.

Current smoking status and practice, such as the frequency of smoking, may influence the behavioral response to a price increase^28^. These variables were assessed by self-report and captured the frequency and quantity of smoking, and smoking history. A current smoker was defined as someone who smoked cigarettes at the time of interview. Current smokers comprised both daily smokers and occasional smokers (those who smoked on some days)^22^. Cigarette consumption distinguished three levels: very heavy (>20 cigarettes per day), heavy (10–19 cigarettes per day) and light (<10 cigarettes per day). Even though historically, Chinese smokers have largely been able to mitigate the impact of price rises, the price of cigarettes that are usually purchased may be sensitive to the price increase imposed by the 2015 tax^28^. Current expenditure on cigarettes was measured by the question: ‘How much does a packet of the cigarettes you usually smoke cost?’. Response options were: <5; 5–9; 10–14; and >15 RMB.

Health status might also influence the decision to purchase cigarettes under changing price conditions, although research in China shows an unclear relationship between levels of smoking and self-assessed health status^29^. Self-assessed health status was measured by the question: ‘How do you assess your health status?’. Response options were: ‘very good’; ‘good’; ‘usually good’; ‘poor’; and ‘very poor’.

### Statistical analysis

All data were entered into a database using Microsoft Excel. The dataset was then imported into SAS (9.4 version) for the statistical analyses. Descriptive statistics were calculated for PSR and all other variables. Both unadjusted and adjusted methods were used in the analyses. The unadjusted method incorporated all explanatory and control variables as independent variables in the analysis. The adjusted method considered the influence of potentially confounding factors as covariates in the multivariable logistic models. Five models were created to explore associations between explanatory factors and PSR following the 2015 tax increase: Model 1 incorporated sociodemographic variables; Model 2 incorporated the measure of household smoking restrictions; Model 3 incorporated the measure of workplace smoking restrictions; Model 4 incorporated anti-smoking information environment exposure; and Model 5 covered all independent variables. All tests were two-tailed with statistical significance set at p<0.05.

Data analysis was carried out in SAS 9.4. Community was used as the clustering unit to account for within-clustering correlation. Weighted analysis methods were: 1) sampling weights, as the inverse of the probability of selection, calculated at city and district-level, and then multiplied together; 2) non-response weights, comprising household and individual aspects; and 3) post-stratification weights, calculated using age (<25, 25–34, 35–44, 45–54, and >55 years)^22,30^. The final overall weights were computed as the product of the prior weights.

## RESULTS

Table 1 shows the characteristics of the sample, and prevalence of PSR by category. Specifically, a total of 6500 male individuals were identified as potential subjects for this study. Of these, 6010 (93.9%) were contacted and agreed to participate in the survey. Of the 6010 questionnaires, 5782 provided a valid response^22^. Of the 5782 participants, 2852 were smokers – a prevalence of 44.8% (95% CI: 41.1–48.5)^22^. The study sample comprised all smokers (n=2852). When asked if they had reduced their cigarette consumption in response to the 2015 tax increase, 74.8% of the sample indicated no change, with an additional 19.0% (n=582) indicating that their smoking had decreased a little, 6.2 % (n=167) indicated that their smoking had decreased moderately, with 2.6% (n=66) reporting a greater reduction, 2.7% (n=77) reporting ‘a large reduction’, and 0.9% (n=24) they had quit smoking. Overall, 25.2% (95% CI: 22.5–27.9) reduced their smoking; 3.5% (n=95) of respondents thought that cigarettes are currently cheap, 7.8% (n=234), 52.1% (n=1403), 27.9% (n=813), and 8.7% (n=307), respectively, reported that the cigarette price was cheap, neutral, expensive, and very expensive.

**Table 1:** Characteristics of participants and prevalence of PSR by category

*Characteristics*	*n*	*%*	*Prevalence*	*OR (95% CI)*
**Age** (years)				
<25 (Ref.)	325	11.9	19.5	1
25–34	669	21.8	22.9	1.23 (1.04–1.45)*
35–44	734	20.3	27.4	1.56 (1.19–2.05)**
45–54	662	22.5	25.8	1.44 (1.21–1.70)**
≥55	462	23.4	27.8	1.59 (1.22–2.08)**
**Ethnicity**				
Han (Ref.)	2725	96.3	24.9	1
Minority	127	3.7	32.2	1.43 (0.81–2.51)
**Education level**				
Elementary school or lower (Ref.)	238	14.0	30.5	1
Junior high school	733	29.3	23.3	0.69 (0.54–0.88)**
High school	896	26.2	25.3	0.77 (0.64–0.94)**
Junior college	590	17.6	24.8	0.75 (0.55–0.97)*
College	395	12.9	24.2	0.94 (0.75–1.17)
**Marital status**				
Unmarried (Ref.)	656	23.1	23.8	1
Married	2055	72.0	25.8	1.11 (0.90–1.37)
Divorced or widowed	141	4.9	22.8	0.95 (0.68–1.32)
**Occupation**				
Managers and service workers (Ref.)	992	33.0	24.7	1
Professionals	240	8.5	22.5	0.88 (0.60–1.30)
Operations	829	38.6	26.7	1.12 (0.71–1.76)
Retired	259	11.7	25.9	1.07 (0.39–2.91)
Other	532	18.2	24.4	0.98 (0.67–1.45)
**Annual income** (RMB)				
<20000 (Ref.)	780	28.6	30.0	1
20000–39999	850	28.6	25.3	0.80 (0.67–0.94)**
40000–49999	547	18.4	24.4	0.76 (0.57–0.98)*
≥50000	675	24.3	20.2	0.60 (0.34–0.97)*
**Cigarettes smoked per day**				
<10 (Ref.)	853	26.6	34.1	1
10–19	1008	32.6	27.8	0.77 (0.31–1.82)
≥20	991	40.0	17.3	0.40 (0.34–0.49)**
**Smoking frequency**				
Daily (Ref.)	2168	76.1	20.9	1
Occasionally	684	23.9	38.9	2.41 (2.18–2.67)**
**Cigarette price** (RMB/pack)				
<5 (Ref.)	166	4.8	36.7	1
5–9	719	21.9	32.2	0.82 (0.598–1.16)
10–14	1119	37.7	23.6	0.53 (0.31–0.90)*
≥15	848	35.5	21.1	0.46 (0.42–0.51)**
**Health status**				
Very good (Ref.)	866	31.4	22.4	1
Good	1072	37.0	24.5	1.12 (0.97–1.30)
Usually good	755	25.6	29.1	1.41 (1.22–1.65)**
Poor or very poor	139	6.0	27.9	1.34 (1.03–1.75)*
**Smoking in home**				
Unlimited (Ref.)	1105	41.9	21.3	1
Limited in some areas	1033	35.2	26.0	1.28 (1.08–1.54)**
Limited throughout	714	22.8	31.2	167 (1.47–1.92)**
**Smoking in workplace**				
Unlimited (Ref.)	961	36.5	22.1	1
Limited in some areas	1007	33.7	25.8	1.58 (0.87–1.18)
Limited throughout	884	29.8	28.2	2.43 (1.05–4.02)*
**Anti-smoking advertising exposure in last 6 months**				
0 (Ref.)	990	32.4	21.9	1
1	536	16.1	26.6	1.29 (1.02–1.65)*
2	476	18.6	26.3	1.27 (1.10–1.48)**
≥3	847	33.0	28.3	1.42 (1.20–1.89)**

RMB: 1000 Chinese Renminbi about US$150 (average 2016).

* p<0.05.

** p<0.01

Those respondents on lower incomes were significantly more likely to have reported that they had reduced their smoking in response to the price rise, as were respondents aged >35 years and those reporting only ‘generally’, ‘poor’ or ‘very poor’ health status. Education, marital status, and occupation demonstrated no systematic trend or significant association with PSR.

Smoking practices among the sample are shown in Table 1, with 26.6% of respondents reporting they smoked <10 cigarettes per day, 32.6% smoked 10–20, and 40% smoked ≥20 per day. Those smoking ≥20 cigarettes per day were significantly less likely to report reducing their consumption in response to the price increase. Occasional smokers were significantly more likely to report that they had reduced smoking compared with daily smokers. The majority (73.2%) of the study group paid ≥10 RMB for their pack of cigarettes, with those paying more being less likely to reduce consumption due to the price increase.

The three environmental independent variables were also reported in the unadjusted analyses: smoking restrictions in the home; smoking restrictions at work; and anti-smoking information environment exposure. A relatively high proportion of respondents (58%) reported some or total restrictions at home, which was significantly associated with reduced consumption in response to increased price. Similarly, workplace restrictions on smoking were significantly more likely to be associated with smoking reduction in response to an increase. With respect to exposure to an anti-smoking information environment, two-thirds of respondents had very limited exposure: none (32.4%), once (16.1%), twice (18.6%), and three times or more (28.3%). In the unadjusted model, there was more trend in the decision to reduce smoking in response to the tax increase relative to the amount of reported exposure to an anti-tobacco information environment.

In the multiple logistic regression analysis (Table 2), most relationships between variables did not change from those manifesting for the unadjusted model. Only core sociodemographic variables (age, education, and income) were entered into Model 1, with education dropping out, as in the unadjusted analysis. Age and income persisted in Models 2–5, with similar odds ratios and high levels of statistical significance across most categories. Models 2–4 incorporated individual environmental restrictions. Model 2 incorporated smoking restrictions in the home, with a greater likelihood of PSR when restrictions occurred either throughout or in part of the home, compared with no restrictions. Respective adjusted odds ratios were 1.33 (95% CI: 1.07–1.67) and 1.67 (95% CI: 1.32–2.80) for partial and total restriction. Model 3, which incorporated workplace smoking restrictions, showed an association with total restriction 1.43 (95% CI: 1.09–1.85). Model 4 included a measure of exposure to an anti-smoking information environment. A single exposure showed no association with PSR, but there was an association with two (AOR=1.32; 95% CI: 1.15–1.52) and three plus exposures (AOR=1.39; 95% CI: 1.10–1.79), respectively. Workplace restrictions dropped out in Model 5, the full model containing all three environmental variables.

**Table 2:** Results from multiple logistic regression analysis

*Variables*	*Model 1 Demographic and personal variables AOR (95% CI)*	*Model 2 Restricted smoking in the home AOR (95% CI)*	*Model 3 Restricted smoking in the workplace AOR (95% CI)*	*Model 4 Anti-smoking information environmental exposure model AOR (95% CI)*	*Model 5 Full model AOR (95% CI)*
**Age** (years)					
<25 (Ref.)	1	1	1	1	1
25–34	1.38 (1.17–1.62)**	1.35 (1.20–1.53)**	1.40 (1.18–1.65)**	1.38 (1.16–1.63)**	1.38 (1.21–1.57)*
35–44	1.80 (1.39–2.33)**	1.81 (1.45–2.25)**	1.86 (1.40–2.47)**	1.81 (1.39–2.37)**	1.86 (1.50–2.23)**
45–54	1.81 (1.47–2.23)**	1.85 (1.61–2.13)**	1.89 (1.55–2.31)**	1.80 (1.44–2.73)**	1.94 (1.60–2.39)**
≥55	1.90 (1.48–2.43)**	1.88 (1.53–2.30)**	2.00 (1.56–2.57)**	1.93 (1.49–2.50)**	2.00 (1.56–2.57)**
**Annual income** (RMB)					
<20000 (Ref.)	1	1	1	1	1
20000–39999	0.74 (0.62–0.87)**	0.74 (0.62–0.88)**	0.73 (0.61–0.87)**	0.74 (0.62–0.88)**	0.74 (0.61–0.87)**
40000–49999	0.63 (0.53–0.76)**	0.62 (0.51–0.75)**	0.62 (0.52–0.73)**	0.63 (0.52–0.76)**	0.66 (0.53–0.83)**
≥50000	0.55 (0.32–0.94)*	0.53 (0.29–0.95)**	0.53 (0.30–0.92)*	0.55 (0.32–0.96)*	0.53 (0.29–0.94)*
**Cigarettes smoked per day**					
<10 (Ref.)	1	1	1	1	1
10–19	0.87 (0.79–0.97)**	0.88 (0.80–0.96)**	0.87 (0.79–0.99)*	0.87 (0.79–0.95)**	0.86 (0.79–0.94)**
≥20	0.46 (0.38–0.57)**	0.48 (0.40–0.58)**	0.48 (0.40–0.57)**	0.46 (0.38–0.57)**	0.46 (0.38–0.56)**
**Smoking frequency**					
Daily (Ref.)	1	1	1	1	1
Occasionally	1.99 (1.81–2.20)**	1.98 (1.83–2.15)**	1.96 (1.79–2.15)**	1.95 (1.81–2.15)**	1.93 (1.80–2.07)**
**Cigarette price** (RMB/pack)					
<5 (Ref.)	1	1	1	1	1
5	0.5 (0.52–1.08)	0.76 (0.53–1.08)	0.76 (0.54–1.09)	0.74 (0.49–1.10)	0.75 (0.50–1.11)
10–14	0.48 (0.22–1.05)	0.47 (0.22–0.99)*	0.47 (0.22–1.03)	0.46 (0.19–1.09)	0.49 (0.19–1.03)
≥15	0.40 (0.29–0.57)**	0.41 (0.30–0.55)**	0.40 (0.29–0.55)**	0.39 (0.26–0.58)**	0.39 (0.27–0.56)**
**Health status**					
Very good (Ref.)	1	1	1	1	1
Good	1.02 (0.85–1.22)	1.03 (0.86–1.23)	1.02 (0.85–1.22)	1.03 (0.85–1.24)	1.03 (0.87–1.23)
Usually good	1.38 (1.05–1.82)*	1.42 (1.08–1.86)*	1.39 (1.04–1.86)*	1.41 (1.05–1.88)*	1.44 (1.09–1.91)*
Poor or very poor	1.09 (0.89–1.33)	1.17 (0.96–1.44)	1.11 (0.92–1.34)	1.09 (0.86–1.37)	1.18 (0.90–1.53)
**Smoking in home**					
Unlimited (Ref.)		1			1
Limited in some areas		1.33 (1.07–1.67)**			1.35 (1.08–1.70)*
Limited throughout		1.67 (1.32–2.08)**			1.67 (1.32–2.17)**
**Smoking in workplace**					
Limited throughout (Ref.)			1		
Limited in some areas			1.12 (0.86–1.47)		
Unlimited			1.43 (1.09–1.85)*		
**Anti-smoking advertising exposure in last 6 months**					
0 (Ref.)				1	1
1				1.20 (0.98–1.49)	1.16 (0.93–1.43)
2				1.32 (1.15–1.52)**	1.30 (1.13–1.50)**
≥3				1.39 (1.10–1.79)**	1.41 (1.11–1.82)**

AOR: adjusted odds ratio. RMB: 1000 Chinese Renminbi about US$140.

* p<0.05.

** p<0.01.

## DISCUSSION

This study was undertaken one year after the 2015 cigarette tax increase in China. The findings show that about one in five urban male smokers in our sample reported a reduction in smoking due to the increase, and 6.2% reported a high reduction (including the 0.9% who reported quitting). This finding is notable given the modest size of the tax increase. After the national 2015 cigarette tax, the excise tax represents 36.3% of the cigarette price, which is significantly lower than the World Health Organization’s recommended level of 70%^14^. Only one-third of our respondents deemed cigarette prices as expensive, indicating that the current price of cigarettes does not inhibit the smoking of the large majority of urban male smokers.

Our study also found that older populations show higher sensitivity to the price measures. This excess may be due to their having more health problems and health awareness than younger counterparts^31^. Household income was negatively associated with PSR. This aligns with economic theory. Empirical research has also found that good economic conditions make people less sensitive to price measures^7,11^. Occasional smoking was positively associated with PSR. This may be related to nicotine dependence, where greater dependence impedes behavioral change^32^. Occasional smokers have lower nicotine dependence than daily smokers, so they can more easily modify their behavior. Our study found that both cigarette quantity and cigarette price were positively associated with higher PSR. These findings may relate to consumer burden, where the demand for cigarettes was influenced by cigarettes price and the quantity consumed^30^.

We also found that exposure to an anti-smoking information environment, beyond just a single exposure, is associated with PSR among urban Chinese males. This relationship may be explained by motivation–behavioral skills and health belief theory^21^. With reinforcement, an anti-smoking information environment may increase smoker awareness of the adverse health effects of smoking and desire to reduce smoking. In turn, this may make smokers more sensitive to rises in cigarette prices. In common with findings for many other middle income and lower countries, overall public awareness of the hazards of smoking is low in China^28^. This lack of awareness reduces the ‘readiness’ to limit consumption in response to higher prices, and encourages smokers to respond in alternative ways, such as switching to cheaper brands^28^. Using mass media and other approaches to disseminate information about the adverse health effects of smoking will increase understanding, help stigmatize smoking, and encourage readiness of smokers to reduce consumption in the face of rising prices^33^.

In our study, the presence of both home and workplace environmental restrictions also appeared important in inducing PSR among urban Chinese males following the 2015 tax increase. Since this restriction is largely voluntary, it is a particularly powerful indicator of a denormalization trend in smoking. Denormalized settings will enhance sensitivity to any ‘trigger’ to reduce consumption, including increased price^34^. This finding likely reflects changing social norms and improvements in tobacco control awareness and beliefs due to the introduction of smoke-free workplaces; they promote greater sensitivity to cigarette pricing^28,35^.

Eriksen and Cerak^35^ argue that the combined impact of these environmental restrictions and anti-smoking information can promote the denormalization of smoking. The mechanism is likely the combined impact of these environmental restrictions and an anti-smoking information environment on the denormalization of smoking^35^. Our study showed that both an anti-smoking information environment and environmental smoking restrictions were positively associated with PSR. It is important to embrace these measures in a cigarette tax policy to improve PSR synergy. These findings indicate that government tobacco policy will be more effective if it incorporates sound anti-smoking information and restrictive smoking environments, as well as a price measure. Overall, the price elasticity of cigarette smokers in China is lower than in some other countries^6,10,36^. China should implement strong national advocacy for tobacco control and a smoke-free policy to create a 100% smoke-free environment in order to enhance sensitivity to an increase in excise taxes among smokers.

### Strengths and limitations

This study has strengths and limitations. It is the first study to examine anti-tobacco environmental impact upon smoker PSR. The cross-sectional study design is an important limitation since it precludes causal inference. On the other hand, we employed a nationwide sample. Future studies need to compile longitudinal surveillance data to examine the anti-tobacco environmental impact upon smoker PSR. A second limitation is that only male urban residents were included in our survey. Consequently, our results are not generalizable to the overall Chinese population, which includes its very large rural populace. Because of the modest increase in price, associated with the 2015 tax increase, we could not ignore the likelihood that other factors might influence PSR, a third study limitation. Further study is necessary. A fourth limitation is that PSR is based on self-report. Our questions were informed by cigarette price theory, which posits that a price increase induces smoking reduction. However, this may produce suggestion bias, which in turn may cause overestimation of the outcome. Indeed, it may be less suggestive just to have asked about the change in cigarette consumption during the period. However, comparative research indicates that Chinese respondents are most honest in answering questions about smoking^31^. Thus, any suggestion bias is likely modest, but we cannot accurately estimate its size in this study, with further study necessary. A final limitation concerns sample representativeness. Our sample was drawn from six large cities, and thus they may not represent all cities in China. However, to achieve a high degree of large city representation in our sample, we were careful to utilize criteria adopted in previous studies, and to select cities from different geographical regions with variable economic development. Our survey was large-scale and the study results are likely a strong indicator of the extent and degree of PSR-related issues within the Chinese urban population.

## CONCLUSIONS

This study provided confirmation that increased exposure to an anti-smoking information environment and smoking restrictions in the home and workplace are positively associated with PSR. It adds substantially to understanding PSR among urban Chinese male smokers. Findings indicate that implementing environmental smoking restrictions and public health education about tobacco control may increase the price sensitivity of smokers. As recommended by the World Health Organization in its report on tobacco pricing, gains from tobacco control policies occur when multiple policies are implemented in concert and are mutually reinforcing^1^. This study suggests that larger increases in cigarette prices are necessary to challenge the purchasing decisions of smokers in China, and that environmental smoking restrictions and public education campaigns must be strengthened to increase the impact of price increase as a tobacco control measure.

## Data Availability

The data that support the findings of this study are available on request from the corresponding author. The data are not publicly available due to privacy or ethical restrictions.
